# Effect of *Salvia officinalis* on diabetic patients

**DOI:** 10.12861/jrip.2013.18

**Published:** 2013-06-01

**Authors:** Saeed Behradmanesh, Fatemeh Derees, Mahmoud Rafieian-kopaei

**Affiliations:** ^1^Department of Internal Medicine, Shahrekord University of Medical Sciences, Shahrekord, Iran; ^2^Medical Plants Research Center, Shahrekord University of Medical Sciences, Shahrekord, Iran

**Keywords:** *Salvia officinalis*, Sage, Diabetic nephropathy

## Abstract

**Introduction:** Herbs are rich sources of natural antioxidants, and are used in traditional medicine for the control and treatment of many diseases. The reducing effect of a large number of these plants on blood glucose has been approved in animal models and clinical studies.

**Objectives:** This study was therefore, performed to investigate the hypoglycemic effect of *Salvia officinalis* on blood glucose, Glycosylated hemoglobin (HbA1c), lipid profile, liver and kidney function tests.Patients and Methods: A double-blind clinical trial was carried out on 80 type II diabetic patients who had not reached the ideal control of the disease. Patients were randomly divided into two equal groups of case and control. The case group received *Salvia officinalis* and the control group received placebo tablets three times a day for three months. The fasting blood sugar (FBS) and 2 hours postprandial (2hpp) glucose were checked at the beginning and every 2 weeks, for three months Glycosylated hemoglobin (HbA1c), lipid profile, liver and kidney function tests were also measured at the beginning and at the end of trial and compared in two mentioned groups.

**Results:** The 2hpp blood sugar and cholesterol levels were significantly decreased in *Salvia officinalis* treated patients compared to control group (p<0.05). There were no significant changes in glycosylated hemoglobin and FBS between the two groups.

**Conclusion:** Results showed that *Salvia officinalis* might be beneficial in diabetic patients to reduce 2hpp and cholesterol. However higher doses might be needed to decrease fasting blood glucose and glycosylated hemoglobin.

**Implication for health policy/practice/research/medical education:**

Results showed that *Salvia officinalis* might be beneficial in diabetic patients to reduce 2 hours postprandial glucose and cholesterol. However higher doses might be needed to decrease fasting blood glucose and glycosylated hemoglobin.

## Introduction

Diabetes mellitus is considered as the most common endocrine disease, the prevalence of which is being increased in the human population (1). The metabolic aspect of diabetes is characterized by moderate to severe hyperglycemia and impaired metabolism of nutrients including proteins, carbohydrates and lipids (2). The side effects of taking insulin and oral hyperglycemic agents have brought about a growing interest among this group of patients for using natural products having anti-diabetic activity (3). Herbs are rich sources of natural antioxidants, and are used in traditional medicine for the control and treatment of many diseases. The reducing effect of a large number of these plants on blood glucose has been approved in animal models and clinical studies (4). Studies on animals have shown that more than 400 plant species have hypoglycemic activity and several laboratories are isolating edible herbal hypoglycemic compounds (5). One of these plants is *Salvia officinalis* (6).

*Salvia officinalis* has numerous common names including sage, common sage, garden sage, golden sage, true sage, culinary sage, kitchen sage, Dalmatian sage, and broadleaf sage. *Salvia officinalis* is a member of Lamiaceae family and native to the Mediterranean region, however it has naturalized in many regions throughout the world. It has a long history of medicinal and culinary uses (7).

*Salvia officinalis* one of the essential herbs with a savoury and slightly peppery flavor. Essential oil of sage contains cineole, borneol, and thujone. Sage leaf contains tannic acid, oleic acid, ursonic acid, ursolic acid, niacin, nicotinamide, flavones, flavonoid glycosides, cornsole, cornsolic acid, fumaric acid, chlorogenic acid, caffeic acid, and estrogenic substances (8). Investigations have taken place into using sage as a treatment for hyperlipidemia and Alzheimer’s disease (8,9).

Antioxidants affect blood sugar and antioxidant properties of or *Salvia officinalis* L (Sage) leaves are known (7-9). In this study, 150 mg *Salvia officinalis *tablets (three times per day) were studied on blood glucose, Glycosylated hemoglobin (HbA1c), lipid profile, liver and kidney function tests.

## Patients and Methods

### Patients

In a randomized clinical trial 80 patients with type-2 diabetes who had not any complications of diabetes (based on description of their medical situation, physical examination, and paraclinical findings) including retinopathy, nephropathy and cardiovascular diseases, and those who had not achieved ideal control of diabetes and were willing to participate in the study entered and were divided into two equal groups.

The project was done with conservation of ethics and obtaining license from the ethics committee of Shahrekord University of Medical Sciences and obtaining the written consent of participants. Patients received *Salvia officinalis* tablets (150 mg extract) or placebo 3 times a day.

Patients continued receiving their anti-diabetic drugs and other oral medications. First, a questionnaire containing information on age, sex, weight, blood pressure, family history of diabetes and duration of the disease was completed. The treatment period was three months and at the beginning and the end of the study the Glycosylated hemoglobin and lipid profile as well as liver and kidney function tests were taken. Laboratory tests at the beginning of the study and every 2 weeks, fasting blood sugar (FBS) and postprandial sugar were checked. Moreover, indicators relating to drug tolerance and the drugs’ side effects were evaluated.

Patients were instructed to follow their type of diet and daily activities during the course of study. These factors were controlled at the two-week visits as well. Moreover, the symptoms of hypoglycemia and the tasks needed for its treatment were taught. After the end of the 3-month period patients repeated the tests taken at their arrival into the study.

### Ethical issues

(1) The research followed the tenets of the Declaration of Helsinki; (2) informed consent was obtained; (3) the research was approved by ethical committee of Shahrekord University of Medical Sciences.

### Statistical analysis

Results were analyzed using paired and independent *t*-tests and repeated measures ANOVA.

## Results

Out of 80 patients who were evaluated, 19 patients were men and 51 were women. Five patients of each group were excluded due to uncontrolled high blood sugar, need for insulin injection, hospitalization and lack of proper cooperation. On the other hand, two patients in the drug group showed mild gastrointestinal complications without stopping the drug use.

Table 1  illustrates the mean and SD of the variables including age, body mass index (BMI) and duration of the disease in drug and control groups, in which none of them had significant difference.

**Table 1:** Mean and standard deviation (SD) different variables in the groups under study

**Statistical Index→** **Variable↓**	**Drug**	**Placebo**
	Mean±SD	Mean±SD
**Age** (Year)	53.2±10.4	51.7±9.9
**BMI** (kg/m^2^)	27.8±4.0	29.5±4.2
**Duration of the disease**	69.9±62.3	74.4±57.7
p>0.05 between 2 groups		

Glycosylated hemoglobin value were respectively 7.9±0.6 and 7.8±0.6 in drug and placebo groups at the beginning of the study (p>0.05) and were respectively 7.43±0.75 and 7.51±0.70 (p>0.05), at the end of the study.

Table 2  shows the comparison between the mean concentration of fasting blood sugar (FBS) and 2-hour postprandial blood glucose (2hpp) at the beginning and every two weeks up to three months. The results show a significant decrease in 2-hour postprandial blood glucose (2hpp) in the drug group compared to the placebo group.

**Table 2:** The comparison of groups for fasting blood sugar (FBS) and 2 hour postprandial blood glucose (2hpp) in different times

**Statistical Index→** **Blood Factor↓**		**On arrival**	**2** ^nd^ ** week**	**4** ^rd^ ** week**	**6** ^th^ ** week**	**8** ^th^ ** week**	**10** ^th^ ** week**	**12** ^th^ ** week**
		Mean±SD	Mean±SD	Mean±SD	Mean±SD	Mean±SD	Mean±SD	Mean±SD
**FBS** (mg/dl)	drug	144.2±43.6	140.7±30.3	145.34±33.0	140.4±28.2	133.3±38.6	129.2±35.5	115.0±45.9
	Placebo	142.9±34.7	138.6±29.8	137.02±32.5	133.2±28.0	140.0±31.5	141.5±34.5	143.4±39.5
**BS2hpp** (mg/dl)	drug	222.0±58.4	194.251.0	196.20±51.4	182.7±39.5	181.4±37.0	185.9±22.0	174.5±21.0*
	Placebo	214.6±42.8	196.6±45.3	198.80±48.1	194.2±42.7	201.0±41.3	191.8±43.4	207.7±49.2
FBS or BS2hpp was not different during various weeks but *in 12^th^ week BS2hpp in drug group was less than placebo group (p<0.05).								

The mean of total cholesterol did not reveal any significant difference at the beginning of the study; however, at the end of study, it was lower in drug group and also lower than its initial value (p<0.05). Table 3  compares the mean triglyceride, total cholesterol, LDL and HDL in the studied groups before and after intervention.

**Table 3:** The comparison between drug and placebo groups before and after intervention.

**Statistical index→** **Blood factor↓**	**Drug**		**Placebo**	
	Mean±SD		Mean±SD	
	Before intervention	After intervention	Before intervention	After intervention
**Triglyceride**	175.1±91.8	180.4±178.5	168.5±84.1	149.74±63.4
**Total cholesterol**	192.4±35.8	101.±39.0*	190.7±29.5	181.62±36.3
**Low density lipoprotein**	110.4±28.7	101.6±31.5	105.3±23.4	102.91±25.6
**High density lipoprotein**	41.4±8.4	42.2±7.6	43.3±8.0	44.02±9.2
* p<0.05, compared with the initiation of the study and placebo group				

Table 4  compares the mean indices of renal function (BUN and creatinine) and liver function (alanine aminotransferase and aspartate aminotransferase) in the drug and placebo groups before and after intervention that showed no significant difference.

**Table 4:** Comparison of blood factors in drug and placebo groups, before and after intervention.

**Statistical index→** **Blood factor↓**	**Drug**		**Placebo**	
	Mean±SD		Mean±SD	
	Before intervention	After intervention	Before intervention	After intervention
**Blood urea nitrogen**	17.9±6.6	17.3±5.6	15.7±6.9	16.2±5.5
**Creatinine**	0.9±0.2	0.9±0.1	0.8±0.2	0.9±0.2
**Alanine aminotransferase**	24.2±9.5	24.8±7.8	24.1±6.9	24.4±6.1
**Aspartate aminotransferase**	22.0±8.2	21.1±6.2	19.4±5.2	20.7±4.6
p>0.05 between 2 groups				

## Discussion

This study was performed to evaluate the hypoglycemic effect of *Salvia officinalis* on blood glucose, Glycosylated hemoglobin (HbA1c), lipid profile, liver and kidney function tests. The fasting blood glucose in drug group was 25 mg/dl less than in control group, however, the difference was not significant (p>0.05). 2-Hour postprandial blood glucose (2hpp) had significant difference in the 12th week of the study (p<0.05).

The lack of significant reduction in fasting blood glucose by drug may be attributed to the of drug’s ineffectiveness on gluconeogenesis and insulin secretion and significant reduction in 2-hour postprandial blood glucose (2hpp) by drug in the twelfth week due to lower insulin resistance.

As mentioned, there was no significant change in glycosylated hemoglobin. Since glycosylated hemoglobin has a considerable correlation with postprandial glucose, the lack of significant result in glycosylated hemoglobin can be attributed to the mean postprandial blood glucose during the treatment which has not been at the desirable level but the reduction in 2-hour postprandial blood glucose (2hpp) at the last week may indicate the fact that if the medication continues, there will be the probability of reduction in glycosylated hemoglobin as well.

The results showed that the mean total cholesterol at the end of the study had significant difference in the drug group compared to the placebo group. Mean triglyceride, LDL and HDL in the studied groups had no significant difference before and after the intervention (p>0.05). The reduction in total cholesterol in the drug group can be an indicator of beneficial effect of this drug on patients who have hyperlipidemia. Fortunately, the drug and placebo have had no adverse and unwanted effect on liver and kidneys that shows the drug’s safety.

In the study conducted by Shafiee-Nick *et al*., the effects of a polyherbal compound, containing six plants (*Allium sativum, Cinnamomum zeylanicum, Nigella sativa, Punica granatum, Salvia officinalis *and * Teucrium polium*) were examined on biochemical parameters in diabetic rats. The diabetic control rats showed further increase in blood glucose after 30 days. Administration of the compound blocked the increase of blood glucose. The six herbal component inhibited the progression of hyperglycemia and decreased serum lipids in diabetic rats. Therefore, it has the potential to be used as a natural product for the management of diabetes (10).

## Conclusion

Considering the mentioned issues, it is possible that not achieving a significant result in the reduction of fasting blood glucose and glycosylated hemoglobin has been due to patients’ disloyalty to diets, insufficient and irregular use of the medication and the low level of effective substances in *Salvia officinalis* tablets. However, one should note their unwanted side effects while using high dosages of herbs. Authors hope that further research be conducted, regarding the mechanism of *Salvia officinalis* effect on blood glucose reduction in diabetic patients, as a considerable help for patients in order to reduce the use of chemical drugs.

## Authors’ contributions

SB designed and performed the research. FD analyzed the data. MRK prepared the final manuscript.

## Conflict of interests

The authors declared no competing interests.

## Ethical considerations

Ethical issues (including plagiarism, misconduct, data fabrication, falsification, double publication or submission, redundancy) have been completely observed by the authors.

The project was registered at IRCT with the code number of IRCT138808202696N1.

## Funding/Support

This paper has been derived from an MD thesis which financially supported by research deputy of Shahrekord University of Medical sciences.
